# Effects of Polyphenols on P-Glycoprotein (ABCB1) Activity

**DOI:** 10.3390/pharmaceutics13122062

**Published:** 2021-12-02

**Authors:** Kuljeet Singh, Szabolcs Tarapcsák, Zsuzsanna Gyöngy, Zsuzsanna Ritter, Gyula Batta, Rosevalentine Bosire, Judit Remenyik, Katalin Goda

**Affiliations:** 1 Department of Biophysics and Cell Biology, Faculty of Medicine, University of Debrecen, 4032 Debrecen, Hungary; singh.kuljeet@med.unideb.hu (K.S.); tarapcsakszabolcs@gmail.com (S.T.); gyongy.zsuzsanna@med.unideb.hu (Z.G.); ritter.zsuzsanna@med.unideb.hu (Z.R.); bagyulajuve@gmail.com (G.B.); rvbosire@med.unideb.hu (R.B.); 2 Doctoral School of Molecular Cell and Immune Biology, University of Debrecen, 4032 Debrecen, Hungary; 3 Utah Center for Genetic Discovery, Eccles Institute of Human Genetics, University of Utah, Salt Lake City, UT 84112, USA; 4 Department of Genetics and Applied Microbiology, Faculty of Science of Technology, University of Debrecen, 4032 Debrecen, Hungary; 5 Institute of Food Technology, Faculty of Agricultural and Food Sciences and Environmental Management, University of Debrecen, 4032 Debrecen, Hungary; remenyik@agr.unideb.hu

**Keywords:** P-glycoprotein (ABCB1), polyphenols, ATPase activity, transport activity, UIC2 reactivity, membrane fluidity, drug-drug interactions

## Abstract

P-glycoprotein (Pgp, ABCB1) is a member of one of the largest families of active transporter proteins called ABC transporters. Thanks to its expression in tissues with barrier functions and its broad substrate spectrum, it is an important determinant of the absorption, metabolism and excretion of many drugs. Pgp and/or some other drug transporting ABC proteins (e.g., ABCG2, MRP1) are overexpressed in nearly all cancers and cancer stem cells by which cancer cells become resistant against many drugs. Thus, Pgp inhibition might be a strategy for fighting against drug-resistant cancer cells. Previous studies have shown that certain polyphenols interact with human Pgp. We tested the effect of 15 polyphenols of sour cherry origin on the basal and verapamil-stimulated ATPase activity of Pgp, calcein-AM and daunorubicin transport as well as on the conformation of Pgp using the conformation sensitive UIC2 mAb. We found that quercetin, quercetin-3-glucoside, narcissoside and ellagic acid inhibited the ATPase activity of Pgp and increased the accumulation of calcein and daunorubicin by Pgp-positive cells. Cyanidin-3O-sophoroside, catechin, naringenin, kuromanin and caffeic acid increased the ATPase activity of Pgp, while they had only a weaker effect on the intracellular accumulation of fluorescent Pgp substrates. Several tested polyphenols including epicatechin, trans-ferulic acid, oenin, malvin and chlorogenic acid were ineffective in all assays applied. Interestingly, catechin and epicatechin behave differently, although they are stereoisomers. We also investigated the effect of quercetin, naringenin and ellagic acid added in combination with verapamil on the transport activity of Pgp. In these experiments, we found that the transport inhibitory effect of the tested polyphenols and verapamil was additive or synergistic. Generally, our data demonstrate diverse interactions of the tested polyphenols with Pgp. Our results also call attention to the potential risks of drug–drug interactions (DDIs) associated with the consumption of dietary polyphenols concurrently with chemotherapy treatment involving Pgp substrate/inhibitor drugs.

## 1. Introduction

Pgp together with several other ABC proteins (e.g., ABCG2, MRP1) possesses a very broad substrate spectrum involving xenobiotics, toxic metabolic side products and numerous chemotherapeutic compounds applied in the treatment of various diseases (e.g., antibiotics, reverse transcriptase inhibitors, antidepressants, narcotics and antineoplastic drugs) (for reviews see [1,2]). Thanks to the expression of Pgp in tissue barriers including the intestinal epithelium, blood–brain, blood–placenta and blood–testis barriers as well as in drug metabolizing and drug extruding organs, such as liver and kidneys, Pgp is an important determinant of the pharmacokinetics of the above-mentioned chemotherapeutic compounds [3,4]. Overexpression of Pgp and/or some other drug transporting ABC proteins (e.g., ABCG2, MRP1) in cancer cells and cancer stem cells often renders the malignant tumors resistant to the different cytotoxic agents used in cancer chemotherapy, leading to the phenomenon of multidrug resistance (MDR) [5,6].

Based on X-ray crystallography data and cryo-electron microscopy Pgp consists of two transmembrane domains (TMDs), forming the drug-binding pocket and the drug translocation pathway, and two intracellular nucleotide binding domains (NBDs), that can collectively bind and hydrolyze ATP [7]. Biophysical and biochemical data suggest that ATP binding to the NBDs induces their dimerization, which propagates to the TMDs, switching them from a high substrate affinity inward facing conformation to a low substrate affinity outward facing conformation making possible the release of substrates, while ATP hydrolysis probably resets the substrate binding conformation [8,9,10,11].

UIC2 is a conformation sensitive monoclonal antibody recognizing a composite epitope transiently formed by the first, third and fourth extracellular loops in the inward-facing conformation of the human Pgp [12,13]. Only 10–30% of Pgps are recognizable for the UIC2 mAb in untreated live cells. However, ATP depletion or treatment of cells with certain inhibitors of Pgp, such as cyclosporine A (CsA), tariquidar, valinomycin, etc., make the rest of the cell surface Pgps available for UIC2 binding [14,15]. The increase in UIC2 reactivity seems to correlate with the inhibitory effect of the compounds, serving as a tool for identifying potent Pgp inhibitors [15,16].

As a transmembrane protein, Pgp is in an intimate relationship with the surrounding plasma membrane. The specific lipid composition as well as the physicochemical properties of the membrane can affect the function of Pgp [17,18].

Polyphenols are secondary metabolites involving over 9000 different compounds that can protect plants from herbivores and pathogens and/or attract pollinators and seed carriers. Polyphenols can be classified into different groups based on their chemical structure, such as phenolic acids, flavonoids, stilbenes and lignans (for reviews see [19,20,21]). Phenolic acids possess very strong antioxidant properties [22]. They can be divided into two subgroups: (1) hydroxybenzoic acids, such as ellagic acid and (2) hydroxycinnamic acids, including caffeic acid, chlorogenic acid and ferulic acid among others. Flavonoids are the most abundant polyphenols in the human diet. They share a common basic chemical structure consisting of two aromatic rings, which are bound together by three carbon atoms that form an oxygenated heterocycle. Based on the different substitution and oxidation state of ring C, flavonoids can be classified into several subclasses, such as flavans (e.g., flavan-3-ols like catechin and epicatechin), flavones (e.g., apigenin, tangeretin, luteolin), flavonols (e.g., quercetin, quercetin-3-glucoside, narcissoside), flavanones (e.g., naringenin), chalcones (e.g., chalconaringenin) and anthocyanidins (e.g., cyanidin-3-O-sophroside (or cyanidin-3,3′-diglucoside), oenin (or malvidin-3-glucoside), malvin (or malvidin-3,5-diglucoside), keracyanin (or cyanidin-3-O-rutinoside) and kuromanin (or cyanidin-3-O-glucoside)).

Consumption of polyphenols has been reported to have many health benefits. Previous laboratory studies as well as clinical trials demonstrated the importance of certain polyphenols in the prevention of carcinogenesis, cardiovascular problems, neurodegenerative diseases and immune disorders. Many of the polyphenols have anti-viral or anti-inflammatory effects [23,24,25,26,27]. Certain fruits and vegetables, such as sour cherry, can be considered as “functional foods” because of their high content of polyphenols with strong antioxidant effects [28].

Previous studies have shown that certain flavonoids and anthocyanins interact with human Pgp, although the data obtained in different experimental systems are often controversial (for reviews see [29,30]). Since polyphenols are present in fruits and vegetables at high concentrations and often used in food supplements or as components of cosmetics, their possible interaction with Pgp may affect the pharmacokinetics of other co-administered Pgp substrate chemotherapeutic drugs.

In this study, we aimed to elucidate the interaction of human Pgp with 15 structurally diverse polyphenols that are abundant in red fruits such as sour cherry (*Prunus cerasus* L.) [31,32,33]. We studied the effects of these compounds on the basal and verapamil-stimulated ATPase activity as well as on the transport activity of Pgp, applying calcein and daunorubicin accumulation assays. In addition, we tested the combined effect of certain flavonoids and a known Pgp substrate chemotherapeutic drug, verapamil. The results of ATPase and transport assays were compared with the effects of the compounds on the conformation of Pgp measured in UIC2 reactivity tests.

## 2. Materials and Methods

### 2.1. Chemicals

All chemicals, cell culture media and supplements used for the study were purchased from Sigma-Aldrich (Budapest, Hungary). Fluorescent dyes including calcein-acetoxymethylester (calcein-AM) and Alexa 647 succinimidyl ester (A647) were obtained from Life Technologies, Inc. (Carlsbad, CA, USA). UIC2, the Pgp-specific mAb, was purified from hybridoma supernatants by affinity chromatography. The antibody-producing hybridoma cell line was obtained from the American Type Culture Collections (Manassas, VA, USA). UIC2 mAb was labeled with A647, and it was purified from the unconjugated dye by gel filtration using a Sephadex G-50 column. The dye-to-protein labeling ratio was determined for every antibody preparation and was found to be around 3. Lipophilic transporter substrates/inhibitors (e.g., calcein-AM and cyclosporine A (CsA)) were dissolved in dimethyl-sulfoxide (DMSO). All tested polyphenols (quercetin, quercetin-3-glucoside, naringenin, ellagic acid, cynidin-3-O-sophroside, oenin, malvin, kuromanin, keracyanin, caffeic acid, chlorogenic acid, trans-ferulic acid, catechin, epicatechin and narcissoside) were HPLC grade (with 99% purity) and were purchased from Sigma-Aldrich (Budapest, Hungary). Polyphenols were dissolved in DMSO or water according to the manufacturer’s instructions. For all experiments, the final DMSO concentration of samples was less than 1% (*v*/*v*).

### 2.2. Cell Lines

The NIH 3T3 mouse fibroblast cell line and its human Pgp overexpressing counterpart, NIH 3T3 MDR1 [34], were kindly provided by Michael Gottesman (National Institutes of Health, Bethesda, MD, USA). Cells were cultured in Dulbecco’s modified Eagle’s medium (DMEM) supplemented with 10% heat-inactivated fetal calf serum (FCS), 2 mM L-glutamine and 0.1 mg/mL penicillin–streptomycin cocktail in humidified atmosphere containing 5% CO_2_ at 37 °C. Cells were regularly tested and were found to be negative for mycoplasma infection using the MycoAlert^®^ mycoplasma detection kit (Lonza Rockland Inc., Rockland, ME, USA).

### 2.3. Membrane Preparations for ATPase Activity Measurements

Cells were harvested by scraping them into phosphate-buffered saline (PBS, pH = 7.4) and washed twice. Membrane preparation was carried out according to Sarkadi’s method [35], with minor modifications. All procedures were carried out at 4 °C. Cell homogenization was performed using a glass-Teflon tissue homogenizer, and crude membrane preparations were prepared in TMEP (50 mM Tris-HCl (pH = 7.0), 50 mM mannitol, 2 mM EGTA, 0.5 mM phenylmethylsulphonyl fluoride (PMSF) and protease inhibitor cocktail (PIC, Sigma-Aldrich, Budapest, Hungary)). Nuclear debris were removed by centrifugation at 500× *g* for 10 min, and the resulting supernatants were centrifuged for 60 min at 28,000× *g*. Finally, the pellet containing cell membranes was re-suspended in TMEP solution and was stored at −70 °C.

### 2.4. ATPase Activity Measurements

The Pgp-specific ATPase activity of the membrane samples was determined by measuring the amount of inorganic phosphate (Pi) released upon ATP hydrolysis in the presence of inhibitors of other major ATPases (NaN_3_ for the F0F1 ATPases, ouabain for Na^+^/K^+^-ATPase and EGTA for the Ca^2+^-ATPases) [35]. Membrane samples (5 μg membrane protein/sample) were pre-incubated with different concentrations of polyphenols with or without 40 µM verapamil in 60 μL ATPase assay premix (50 mM MOPS, 65 mM KCl, 6.5 mM NaN_3_, 2.6 mM DTT, 1.28 mM ouabain, 0.65 mM EGTA, pH = 7.0) in the presence or absence of 100 μM Na_3_VO_4_ (vanadate) for 5 min at 37 °C. Subsequently the ATPase reaction was initiated by the addition of 3.2 mM MgATP. After 25 min incubation at 37 °C, the ATPase reaction was stopped by 40 μL 5% SDS, then the samples were incubated with 105 μL color reagent [36] at room temperature for 30 min. The absorbances were measured at 700 nm using a BioTek Synergy HT plate reader (BioTek Instruments, Winooski, VT, USA), and the amount of released P_i_ was determined in the samples. Since the ATPase activity of ABC transporters is inhibited by vanadate, the Pgp-specific ATPase activity was calculated as the difference of the ATPase activities in the vanadate-treated and untreated sample pairs.

### 2.5. Calcein and Daunorubicin Accumulation Tests

For studying the effect of polyphenols on the transport activity of Pgp, we used the well-established fluorescent Pgp substrates calcein-AM and daunorubicin [37,38]. Harvested cells were washed three times in PBS containing 7 mM glucose (gl-PBS). Subsequently, samples of 0.5 × 10^6^ cells/mL were pre-treated with 10 μM CsA, 40 µM verapamil or the tested polyphenols for 10 min at 37 °C and then further incubated with 3.8 μM daunorubicin for 30 min or 0.5 μM calcein-AM for 20 min. Following three washing steps with ice-cold gl-PBS containing 0.5% FBS, samples were kept on ice until flow cytometry measurement.

### 2.6. UIC2 Reactivity Test

The Pgp overexpressing NIH 3T3 cells (0.5 × 10^6^ mL^−1^ in gl-PBS) were pre-incubated with the tested polyphenols (added at 0–150 µM concentration) or a known Pgp inhibitor CsA (10 µM) for 10 min and then further incubated with 10 μg/mL UIC2-A647 monoclonal antibody at 37 °C. After 30 min of incubation, samples were washed 2 times with ice-cold gl-PBS and centrifuged for 5 min at 435× *g* at 4 °C. The UIC2-A647 fluorescence intensity of the cells was measured by flow cytometry.

### 2.7. Flow Cytometry

Flow cytometry analysis was carried out using a Becton Dickinson FACS Calibur flow cytometer (Becton Dickinson, Mountain View, CA, USA). Calcein, PI and daunorubicin were excited by the 488 nm line of a solid-state laser, and the emitted light was detected using a 502 nm dichroic mirror and a 530/30 nm band-pass filter (for calcein), while the fluorescence of propidium iodide (PI) and daunorubicin was collected applying a 585/42 nm band-pass filter. The fluorescence signal of A647 was detected using a 635 nm red diode laser and a 661/15 nm band-pass filter. Dead cells staining with PI were excluded from the analysis. Fluorescence signals were collected in logarithmic mode, and the cytofluorimetric data were analyzed using the Flowing software (Cell Imaging Core, Turku Centre for Biotechnology, Turku, Finland).

### 2.8. Measurement of Membrane Fluidity

The effects of polyphenols on the membrane fluidity were studied by measuring the steady state anisotropy of 6-Phenyl-1,3,5-Hexatriene (DPH) and 1-(4-Trimethylammonium)-6-Phenyl-1,3,5-Hexatriene (TMA-DPH). Cells (1 × 10^6^ cells mL^−1^ in gl-PBS) were pre-incubated with the tested polyphenols at different concentrations for 10 min at 37 °C and then further incubated with 2 μM DPH or TMA-DPH for 20 min. The fluorescence measurements were carried out at 37 °C without washing the samples using a Horiba Jobin Yvon Fluorolog-3 (Yvon Horiba, Edison, NJ, USA) spectrofluorometer equipped with a thermostated sample holder. Both dyes (DPH and TMA-DPH) were excited at 358 nm and the emitted light was measured at 427 nm. The steady-state fluorescence anisotropy values (*r*) were calculated using the following formula:

$$r=\frac{I_{\mathrm{VV}}-{\mathrm{GI}}_{\mathrm{VH}}}{I_{\mathrm{VV}}+{2\mathrm{GI}}_{\mathrm{VH}}}$$

where, *I_VV_* and *I_VH_* are the vertically and horizontally polarized components of the fluorescence intensities, respectively, both excited by a vertically polarized light. *G* is an instrument-specific correction factor applied for compensating the unequal sensitivity of the detection system for vertically and horizontally polarized light. The fluorescence anisotropy values of DPH and TMA-DPH are inversely proportional to the membrane fluidity [39].

### 2.9. Statistical Analysis

Data are presented as means ± SD. For the statistical analysis of data, SigmaPlot (version 14, SSI San Jose, CA, USA) was used. For the comparison of two samples from normally distributed populations with equal variances, Student’s *t*-test was performed, while in case of unequal variances a Welch’s *t*-test was applied. The assumptions of normality and equal variance were checked by Kolmogorov–Smirnov and Brown–Forsythe tests, respectively. Multiple comparisons were performed with analysis of variance (ANOVA) applying the Holm–Sidak test for post hoc pair-wise comparison of the data. In the case of unequal variances, the Dunnett T3 post hoc pair-wise comparison method was used. Differences were considered significant at *p* < 0.05.

## 3. Results

### 3.1. ATPase Activity Measurements

**Figure 1:**
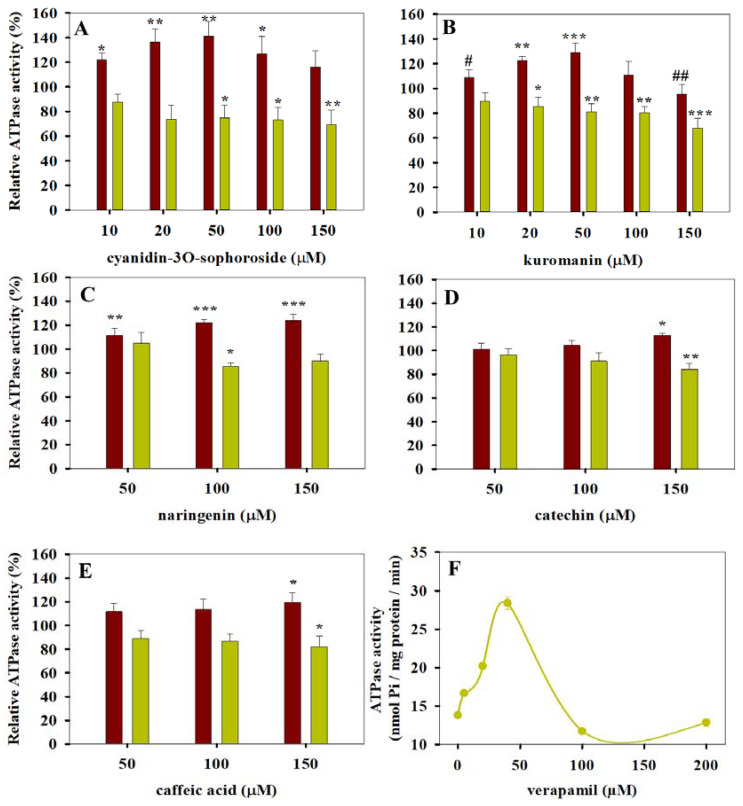
Polyphenols that increase the basal and decrease the verapamil-stimulated ATPase activity of Pgp (**A**–**E**). The effect of polyphenols on the basal and substrate-stimulated ATPase activities of Pgp were measured in the absence (red bars) or in the presence (yellow bars) of 40 µM verapamil, respectively (**A**–**E**). An amount of 40 µM verapamil induced the maximal stimulation of the Pgp-specific ATPase activity in NIH 3T3 MDR1 membrane samples (**F**). Relative Pgp-specific ATPase activities were calculated compared with the polyphenol-untreated samples. Bars represent the means (±SD) of three independent measurements each performed in triplicates. Significant differences compared with the polyphenol-untreated samples are shown by ***: *p* < 0.001, **: *p* < 0.01, *: *p* < 0.05. Significant differences relative to the 50 µM kuromanin-treated cells are indicated by ##: *p* < 0.01, #: *p* < 0.05.

**Figure 2:**
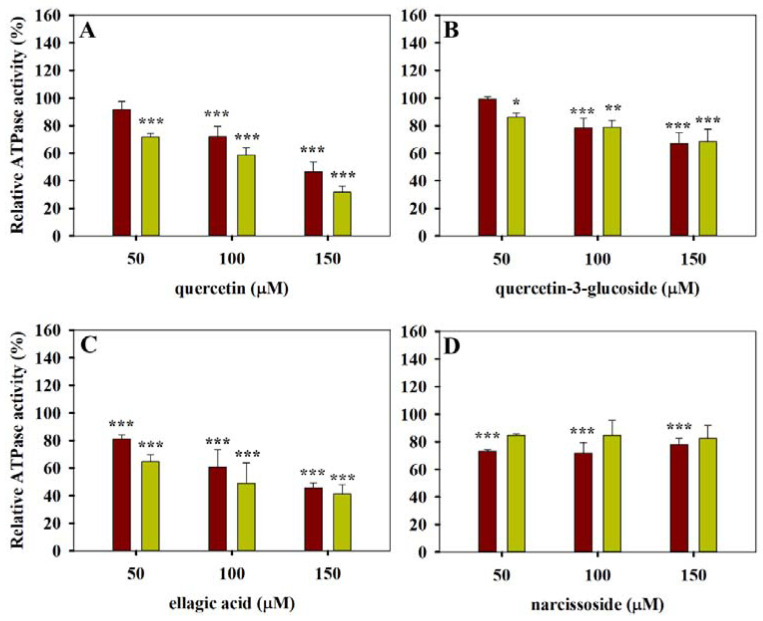
Polyphenols that decrease both the basal and the verapamil-stimulated ATPase activity of Pgp. Basal and substrate-stimulated ATPase activities of Pgp were measured in the absence (red bars) or the presence (yellow bars) of a known Pgp substrate verapamil (40 µM), respectively (**A**–**D**). Relative Pgp-specific ATPase activities were calculated compared with the polyphenol-untreated samples. Bars represent the means (±SD) of three independent measurements each performed in triplicates. Significant differences compared with polyphenol-untreated samples are shown by ***: *p* < 0.001, **: *p* < 0.01, *: *p* < 0.05.

**Figure 3:**
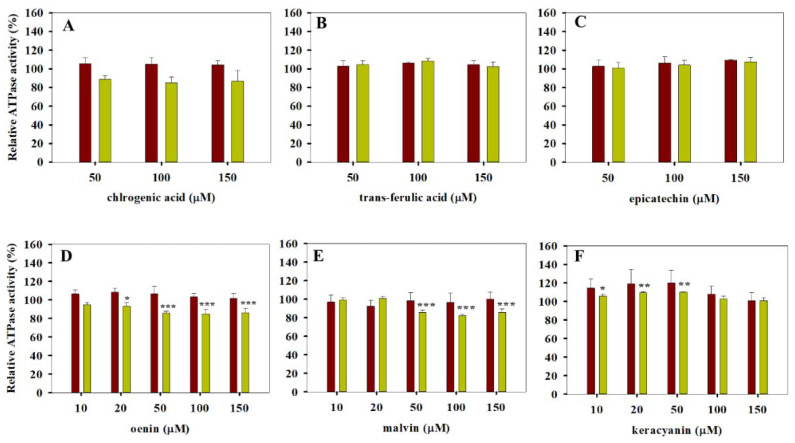
Polyphenols with negligible effect on the ATPase activity of Pgp. Basal and substrate-stimulated ATPase activities of Pgp were measured in the absence (red bars) or the presence (yellow bars) of 40 µM verapamil, respectively (**A**–**F**). Relative Pgp-specific ATPase activities were calculated compared with the polyphenol-untreated samples. Bars represent the means (±SD) of three independent measurements each performed in triplicates. Significant differences compared with polyphenol-untreated samples are shown by ***: *p* < 0.001, **: *p* < 0.01, *: *p* < 0.05.

We measured the effects of 15 dietary polyphenols on the basal and on the verapamil-stimulated ATPase activity of Pgp using membrane samples prepared from NIH 3T3 MDR1 cells (Figure 1, Figure 2 and Figure 3). Due to the high Pgp expression level of the NIH 3T3 MDR1 membrane preparations, a 2- to 3-fold maximal stimulation of the Pgp-specific ATPase activity was observed in the presence of verapamil, a known Pgp substrate (see Figure 1F). Since the highest ATPase stimulation was obtained in the presence of 40 µM verapamil, this concentration was used in all further experiments. Based on the results of our pilot experiments, polyphenols were applied at 10 to 150 μM or 50 to 150 µM concentrations. Based on their behavior in our ATPase activity measurements, the tested polyphenols were divided into three subgroups.

Another group of compounds involving quercetin, quercetin-3-glucoside and ellagic acid strongly decreased both the basal and the verapamil-stimulated ATPase activity of Pgp (Figure 2A–C). Narcissoside had weaker effects without showing concentration dependence (Figure 2D). It seems likely that these compounds are Pgp inhibitors that are not transported by the pump.

On the other hand, the rest of the tested compounds such as chlorogenic acid, trans-ferulic acid, malvin and oenin did not have a statistically significant effect on the basal ATPase activity of Pgp (see Figure 3). Interestingly, epicatechin (Figure 3C) also belongs to this inactive subgroup of polyphenols, while its stereoisomer catechin stimulated the basal activity and decreased the verapamil-stimulated ATPase activity of Pgp when it was applied at 150 µM concentration (see Figure 1D). Oenin and malvin slightly decreased the verapamil-stimulated ATPase activity of Pgp (Figure 3D,E). Generally, the tested polyphenols decreased or did not affect the verapamil-stimulated ATPase activity of Pgp (see Figure 1, Figure 2 and Figure 3) except for keracyanin, which induced a slight increase when it was applied at 10 to 50 µM concentrations (Figure 3F).

### 3.2. Calcein and Daunorubicin Accumulation Experiments

**Figure 4:**
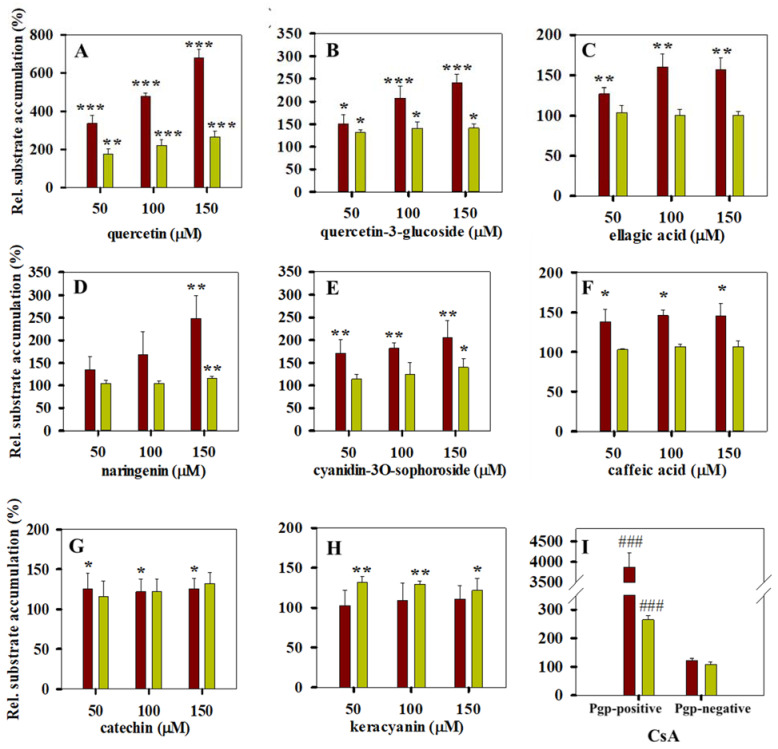
Polyphenols that decrease the transport activity of Pgp in calcein (red bars) and daunorubicin (yellow bars) accumulation experiments (panels (**A**–**H**)). The effect of 10 µM cyclosporine A (CsA) on the calcein and daunorubicin uptake by Pgp-positive and Pgp-negative cells (panel (**I**)). Cells were pre-treated with polyphenols (panels (**A**–**H**)) or CsA (panel (**I**)) for 10 min at 37 °C, and then they were stained with fluorescent Pgp substrates (0.5 µM calcein-AM or 3.8 µM daunorubicin) for 20 min and 30 min, respectively. The intracellular accumulation of calcein and daunorubicin was measured in a flow cytometer. The relative substrate accumulation was calculated by normalizing the mean calcein or daunorubicin fluorescence intensities of the CsA/polyphenol-treated samples to the CsA/polyphenol-untreated control samples and expressed as a percentage (%). Bars represent the means (±SD) of at least three independent measurements. Significant differences compared with the untreated samples are shown by ***: *p* < 0.001, **: *p* < 0.01, *: *p* < 0.05. In panel **I**, significant differences compared with the Pgp-negative cells are shown by ###: *p* < 0.001.

**Figure 5:**
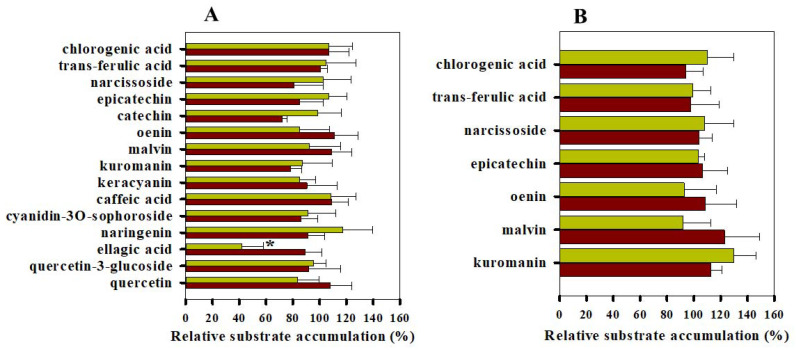
Effect of polyphenols on the calcein (red bars) and daunorubicin (yellow bars) accumulation by Pgp-negative (**A**) and Pgp-positive cells (**B**). Cells were pre-treated with polyphenols applied at 100 µM for 10 min at 37 °C and then further incubated with fluorescent Pgp substrates (0.5 µM calcein-AM or 3.8 µM daunorubicin) for 20 min and 30 min, respectively. The intracellular accumulation of calcein and daunorubicin was measured in a flow cytometer. The relative substrate accumulation was calculated normalizing the mean calcein or daunorubicin fluorescence intensities of the polyphenol-treated samples to the polyphenol-untreated control samples and expressed as a percentage (%). Bars represent the means (±SD) of at least three independent measurements. Significant differences compared with polyphenol-untreated samples are shown by *: *p* < 0.05.

**Figure 6:**
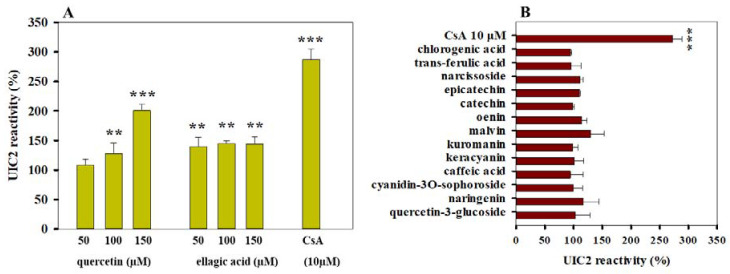
Effect of polyphenols on the UIC2 reactivity of Pgp-positive cells. Quercetin and ellagic acid were tested in the concentration range of 50–150 µM (**A**), while all other polyphenols were applied at 100 µM concentration (**B**). Cells were pre-incubated in the presence or absence of polyphenols or 10 µM cyclosporine A (CsA) for 10 min at 37 °C and then further incubated with 10 µg/mL UIC2-A647 for another 30 min. The UIC2-A647 staining of polyphenol/CsA-treated cells was normalized to that of the untreated cells. Bars represent the means (±SD) of at least three independent measurements. Significant differences compared with untreated samples are shown by ***: *p* < 0.001, **: *p* < 0.01.

To study the effect of the above compounds on the transport activity of Pgp, calcein and daunorubicin accumulation experiments were carried out using the NIH 3T3 (Pgp-negative) and NIH 3T3 MDR1 (Pgp-positive) cell line pair (see Figure 4, Figure 5 and Figure 6). Quercetin had the strongest inhibitory effect on the Pgp-mediated substrate transport, increasing the cellular accumulation of both calcein and daunorubicin in a concentration-dependent manner (Figure 4A). Compared with quercetin, quercetin-3-glucoside had a weaker influence on the transport function of Pgp in accordance with its milder inhibitory effect in our ATPase measurements (compare Figure 4B and Figure 2B). Ellagic acid and naringenin (Figure 4C,D) increased the calcein accumulation to a maximum 200–250% of the control sample, in accordance with literature data [40,41]. At the same time, ellagic acid, naringenin and caffeic acid did not affect the intracellular accumulation of daunorubicin to an appreciable extent (Figure 4C,D,F). Interestingly, keracyanin behaved in the opposite manner, increasing the uptake of daunorubicin by Pgp-positive cells and having no significant effect on the cellular accumulation of calcein (Figure 4H). On the other hand, cyanidin-3O-sophoroside, structurally related to keracyanin (cyanidin-3O-rutinoside), elevated both the calcein and daunorubicin accumulation by Pgp-positive cells (Figure 4E). Catechin also increased slightly the intracellular accumulation of calcein and daunorubicin in accordance with its modest effect on the ATPase activity of Pgp (Figure 4G). In control experiments, 10 µM CsA, a potent inhibitor of Pgp [42,43], strongly increased the uptake of calcein and daunorubicin by Pgp-positive cells, while it did not have any effect on the Pgp-negative cells (Figure 4I).

The polyphenols that were found effective in elevating the daunorubicin and calcein uptake by Pgp-positive cells did not significantly affect the staining of Pgp-negative control cells (Figure 5A), suggesting that the above-described effects are Pgp-specific. The only exception was ellagic acid, which strongly decreased the intracellular fluorescence of daunorubicin in Pgp-negative cells.

All the other compounds including chlorogenic acid, trans-ferulic acid, malvin, oenin and epicatechin were found to be inactive in ATPase assays, and they were also ineffective in substrate accumulation assays (compare Figure 3 and Figure 5B). Interestingly, kuromanin affected both the basal and the verapamil-stimulated ATPase activities of Pgp (Figure 1B), but it did not exhibit significant effects in calcein and daunorubicin assays (Figure 5B).

### 3.3. Effects of Polyphenols on the Conformation of Pgp

Previous studies suggested that efficient Pgp inhibitors may stabilize Pgp molecules in a UIC2 reactive conformational state [16]. Therefore, to further investigate the interaction of the above phytochemicals with Pgp, we also carried out UIC2 reactivity assays. We demonstrated that quercetin induced a significant concentration-dependent increase in UIC2 reactivity (see Figure 6A) in accordance with its strong Pgp inhibitory effect in ATPase and substrate accumulation experiments (Figure 2A and Figure 4A). Ellagic acid also increased the UIC2 reactivity of Pgp-positive cells, although we did not experience concentration-dependence in the tested concentration range (Figure 6A). On the other hand, polyphenols with weaker effects on the ATPase and transport function of Pgp did not affect the UIC2 reactivity of Pgp-positive cells significantly (Figure 6B).

### 3.4. Effects of Polyphenols on Membrane Fluidity

The plasma membrane partitioning and the distribution of polyphenols inside the lipid bilayer of Pgp-positive and Pgp-negative mammalian cells are largely unknown. In order to understand, how polyphenols interact with the plasma membrane and how they affect the membrane fluidity, we performed fluorescence anisotropy measurements using the membrane-specific probes DPH and TMA-DPH. DPH is an apolar molecule that accumulates in the acyl-chain region of the membrane, while TMA-DPH is a positively charged derivative of DPH that prefers the lipid/water interface of the membrane [44]. Therefore, using TMA-DPH and DPH the membrane fluidity and packing order can be estimated in slightly different depths of the plasma membrane since the average fluorophore of TMA-DPH is about 3–4 Å closer to the membrane surface compared with DPH [39,45]. Membrane fluidity and fluorescence anisotropy values of DPH and TMA-DPH show an inverse correlation: higher membrane fluidity results in decreased fluorescence anisotropy values due to the increased rotational and vibrational freedom of the dye in the plane of the membrane, while increased anisotropy corresponds to a decreased membrane fluidity [39].

**Figure 7:**
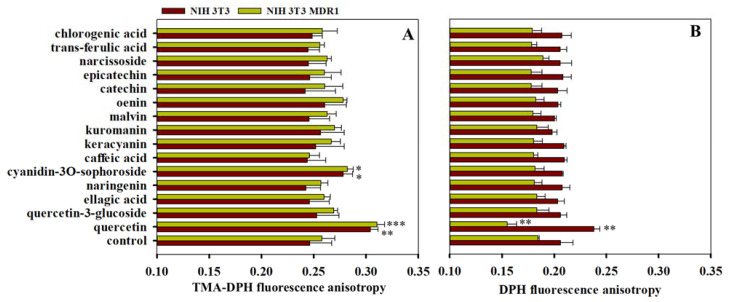
Effects of polyphenols on the steady-state fluorescence anisotropy of TMA-DPH (**A**) and DPH (**B**) in Pgp-positive (yellow bars) and Pgp-negative (red bars) NIH 3T3 cells. Cells were pre-treated with polyphenols applied at 50 µM concentration for 10 min at 37 °C and then further incubated with 2 µM DPH or TMA-DPH for another 20 min. Fluorescence anisotropy values were measured at 37 °C. Bars represent the means ± SD of at least three independent experiments performed in triplicates. Significant differences compared with the polyphenol-untreated control samples are shown by ***: *p* < 0.001, **: *p* < 0.01, *: *p* < 0.05.

The fluorescence anisotropy value of DPH was in the range of 0.18 and 0.20 in untreated Pgp-positive and Pgp-negative NIH 3T3 cells (Figure 7), indicative of high structural order within the membrane, while the fluorescence anisotropy of TMA-DPH varied between 0.24 and 0.26, suggesting lower order in the superficial regions of the membrane. The measured anisotropy values did not differ significantly in the untreated Pgp-positive and Pgp-negative cell lines, in agreement with previous data [46,47]. Quercetin and cyanidin-3O-sophoroside increased the TMA-DPH anisotropy values in both cell lines, indicating a decrease in membrane fluidity at the vicinity of the membrane surface (Figure 7A). Interestingly, in DPH anisotropy measurements, we observed a completely different tendency: quercetin treatment increased the DPH anisotropy in Pgp-negative cells, while a decrease in DPH anisotropy was detected in Pgp-positive cells, suggesting that Pgp activity may affect the intramembrane distribution of quercetin (Figure 7B).

### 3.5. Combined Effects of Polyphenols and Verapamil on Pgp Activity

**Figure 8:**
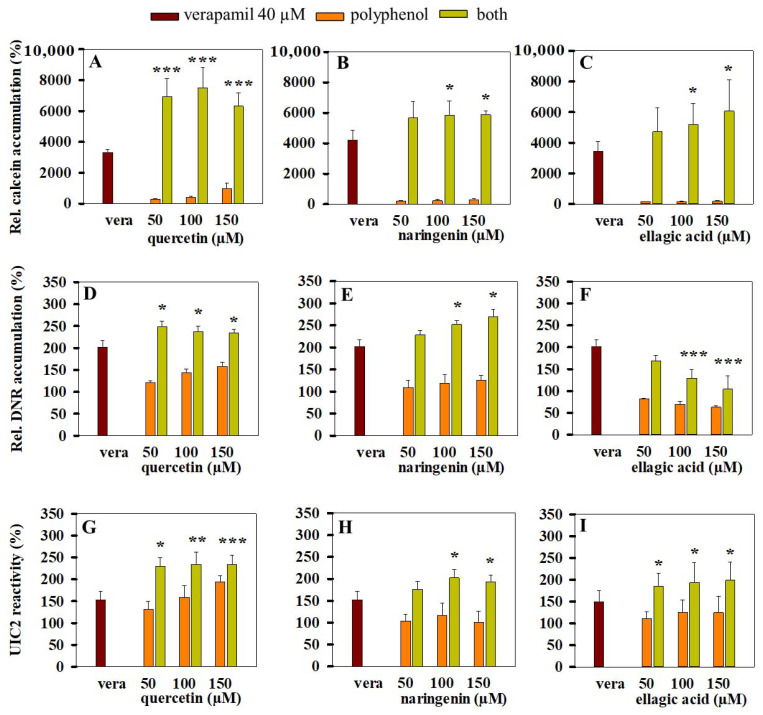
Effects of verapamil–quercetin (**A**,**D**,**G**), verapamil–naringenin (**B**,**E**,**H**) and verapamil–ellagic acid (**C**,**F**,**I**) combinations on the intracellular accumulation of calcein (**A**–**C**) or daunorubicin (**D**–**F**) and on the UIC2 reactivity (**G**–**I**) of NIH 3T3 MDR1 cells. Samples were pre-incubated in the presence or absence of 40 µM verapamil (vera) and/or polyphenols for 10 min at 37 °C and then stained with calcein-AM, daunorubicin or UIC2-A647, as it is described in detail in the Materials and Methods section. Bars represent the means (±SD) of at least three independent measurements. Significant differences compared with the only-verapamil-treated samples are shown by ***: *p* < 0.001, **: *p* < 0.01, *: *p* < 0.05.

We studied the combined effect of structurally distinct polyphenols and verapamil on Pgp activity. The combined addition of 40 µM verapamil and quercetin resulted in higher calcein accumulation compared with the sum of the individual effects of the two compounds, suggesting a possible synergism between them (Figure 8A). On the other hand, an additive effect of the verapamil–quercetin combination was experienced in daunorubicin accumulation assay (Figure 8D). When verapamil was combined with naringenin, additive effects were detected both in calcein and daunorubicin accumulation experiments (Figure 8B,E). Similarly, the combination of verapamil and ellagic acid additively increased the intracellular accumulation of calcein (Figure 8C), while it decreased the daunorubicin uptake compared with the only-verapamil-treated Pgp-positive cells (Figure 8F). Ellagic acid also decreased the staining of Pgp-negative control cells by daunorubicin (see Figure 4I), supporting the idea that Pgp-independent mechanisms are also involved. The decreased intracellular daunorubicin staining in response to ellagic acid treatment is probably explained by previous observations that ellagic acid accumulating in the nucleus may form covalent DNA adducts [48], and thus it may prevent the binding of DNA-specific drugs e.g., daunorubicin. 

The combined application of verapamil and polyphenols facilitated the trapping of Pgps in the UIC2 reactive conformation (see Figure 8G–I).

## 4. Discussion

In the current experiments, we studied the effects of 15 different polyphenolic compounds on the ATPase and transport activities as well as on the conformational state of human Pgp. Here we report for the first time that cyandin-3O-sophroside interacts with Pgp and decreases the membrane fluidity and the packing order in the superficial regions of the membrane. We also observed the Pgp inhibitory effect of ellagic acid and found that it interacts with the chemotherapeutic drug daunorubicin.

Moreover, our experiments proved the previous observations that quercetin is a potent inhibitor of Pgp [49]. In our hands, quercetin strongly decreased both the basal and the substrate-stimulated ATPase activity of Pgp and thus increased the intracellular accumulation of daunorubicin and calcein alike by Pgp-positive cells (see Figure 2A and Figure 4A). When quercetin was applied together with 40 µM verapamil, the two compounds synergistically increased the calcein accumulation of Pgp-positive cells up to the level of Pgp-negative cells (Figure 8A). In simultaneous experiments, we detected a strong increase in UIC2 reactivity in response to the combined quercetin–verapamil treatment, supporting the idea that it stabilizes the UIC2 reactive inward-facing conformation of Pgp molecules (Figure 8G). Although the exact mode of inhibitor binding to Pgp and the action mechanism of potential inhibitors is not known, it seems likely that numerous potent Pgp inhibitors act to stabilize the inward-facing conformational state of the pump [16,50] and consequently hinder the formation of the NBD sandwich dimer and thus inhibit ATP hydrolysis.

We also found that quercetin modifies the TMA-DPH and DPH anisotropy values (see Figure 7), supporting the idea that it can partition into the plasma membrane of live cells and changes the fluidity and the packing order of the membrane either directly or indirectly. Pgp is sensitive to the alterations of the membrane fluidity, and thus the above membrane specific effects of quercetin may also contribute to its Pgp inhibitory effects [17,51]. We observed an increase in TMA-DPH anisotropy in response to quercetin treatment, which reflects the increased ordering of the lipid head groups in agreement with previous experiments carried out on liposomes [52,53].

Interestingly, in DPH anisotropy measurements, we found that quercetin increased the membrane fluidity in Pgp-positive cells, while we observed an opposite change in membrane fluidity in Pgp-negative cells (see Figure 7B). Previous studies suggested that the effect of certain flavonoids (e.g., kaempferol) on membrane fluidity is concentration-dependent, i.e., they decrease membrane fluidity at low concentrations and fluidize the membrane at high concentrations [54]. In accordance with this observation, the above-mentioned fluidizing effect of quercetin in Pgp-positive cells may be explained by the Pgp-dependent sequestration of quercetin in the acyl-chain region of the membrane monitored in DPH anisotropy measurements. However, further experiments are required to understand the fine details of this phenomenon.

We observed that quercetin-3-glucoside possesses weaker Pgp inhibitory effects compared with quercetin (Figure 2B and Figure 4B). At the same time, we found that quercetin-3-glucoside did not affect the TMA-DPH and DPH anisotropy values, implying that it may not reach a membrane concentration sufficient for affecting the packing order and the fluidity of the plasma membrane. The lower membrane partitioning of quercetin-3-glucoside is also supported by its lower octanol–water partition coefficient compared with quercetin [55], although the latter parameter is not always a good predictor for the plasma membrane partitioning of compounds [56]. In addition, the presence of a sugar moiety also changes the shape of the molecule, and thus it may affect its interaction with the drug-binding pocket of Pgp.

We found that certain compounds inhibited the verapamil-stimulated ATPase activity, while they stimulated the basal ATPase activity of Pgp. The stimulatory effect of cyandin-3O-sophroside and kuromanin on the basal ATPase activity of Pgp showed a bell-shaped concentration dependence. A similar tendency was observed for several previously identified Pgp substrates, such as verapamil (see also Figure 1F) or vinblastine [35,57,58]. This concentration dependence is supposed to reflect the gradual saturation of the binding pocket of Pgp with its ligands. The interaction of substrates with Pgp may occur on a modular basis, binding to only one site at lower substrate concentrations, while occupying two or more sites simultaneously at higher concentrations [38]. In agreement with the above explanation, several drugs (e.g., azidothymidine, paliperidone, abacavir) that are efficiently transported by Pgp become a high-affinity inhibitor when two identical molecules are chemically cross-linked, supporting the inhibitory effect of two molecules bound simultaneously to the drug-binding pocket of Pgp [59,60].

Catechin had a weak effect on both the ATPase and the transport activity of Pgp, in agreement with a previous report [61]. In addition, in our current experiments, we also tested epicatechin, a stereoisomer of catechin, and found it completely ineffective. In view of the extremely broad substrate spectrum of Pgp, the discrimination between stereoisomeric compounds seems to be an interesting phenomenon. However, in a previous study, we observed that the recognition of certain retinoids by Pgp and ABCG2 is also stereoselective [47]. Since Pgp and several other ABC transporters take up their substrates/inhibitors directly from the plasma membrane, the selective recognition of these ligands may happen either in the drug-binding pocket or in the plasma membrane.

We also investigated the effect of quercetin, naringenin and ellagic acid in combination with verapamil on the activity of Pgp. We observed that the transport inhibitory effect of the polyphenol–verapamil combinations is additive or synergistic (Figure 8). However, further in vitro and in vivo experiments are required to evaluate the potential significance of polyphenol–verapamil combinations in overcoming the Pgp-mediated multidrug resistance of tumors. On the other hand, these results also call attention to the potential risks of drug–drug interactions (DDIs) associated with the consumption of dietary polyphenols concurrently with chemotherapy treatment involving Pgp substrate/inhibitor drugs. Since a diverse range of polyphenols are proved to be present at high amounts in fruits, vegetables, coffee and tea, even the weak Pgp inhibitory effects of the individual compounds can be amplified by the possible additive or synergistic interactions between them at the level of Pgp.

## 5. Conclusions

Our experiments demonstrated that certain dietary polyphenols present in sour cherry and other red berries are substrates or inhibitors of the human Pgp. When polyphenols were applied alone, they did not provide complete Pgp inhibition. However, different polyphenol–verapamil combinations tested in this study exhibited a very strong Pgp inhibitory effect that might be exploited in chemotherapy protocols for the treatment of multidrug-resistant tumors. On the other hand, the observation that the transport inhibitory effect of polyphenol–verapamil combinations is additive or synergistic supports the potential risk of drug–drug interactions (DDIs) associated with the consumption of dietary polyphenols concurrently with chemotherapy treatment involving Pgp substrate/inhibitor drugs.

## Data Availability

Data obtained or analyzed during the current study are available from the corresponding author upon reasonable request.
